# FXR Activation Accelerates Early Phase of Osteoblast Differentiation Through COX-2-PGE_2_-EP4 Axis in BMP-2-Induced Mouse Mesenchymal Stem Cells

**DOI:** 10.3390/molecules30010058

**Published:** 2024-12-27

**Authors:** Ko Fujimori, Yusuke Iguchi, Yukiko Yamashita, Keigo Gohda, Naoki Teno

**Affiliations:** 1 Department of Pathobiochemistry, Faculty of Pharmacy, Osaka Medical and Pharmaceutical University, 4-20-1 Nasahara, Takatsuki 569-1094, Japan; 2 Faculty of Pharmaceutical Sciences, Hiroshima International University, 5-1-1, Hirokoshingai, Kure 737-0112, Japan; y-iguchi@hirokoku-u.ac.jp (Y.I.); y-yamasi@hirokoku-u.ac.jp (Y.Y.); 3 Computer-Aided Molecular Modeling Research Center, Kansai (CAMM-Kansai), 3-32-302, Tsuto-Otsuka, Nishinomiya 663-8241, Japan; ke.gohda@camm-kansai.org; 4 Graduate School of Pharmaceutical Sciences, Hiroshima International University, 5-1-1, Hirokoshingai, Kure 737-0112, Japan; n-teno@hirokoku-u.ac.jp; 5 Faculty of Clinical Nutrition, Hiroshima International University, 5-1-1, Hirokoshingai, Kure 737-0112, Japan

**Keywords:** mesenchymal stem cells, osteoblasts, FXR, COX-2, PGE_2_

## Abstract

Farnesoid X receptor (FXR), a nuclear receptor, is expressed in calvaria and bone marrow stromal cells and plays a role in bone homeostasis. However, the mechanism of FXR-activated osteoblast differentiation remains unclear. In this study, we investigated the regulatory mechanism underlying FXR-activated osteoblast differentiation using bone morphogenetic protein-2 (BMP-2)-induced mouse ST-2 mesenchymal stem cells. We also synthesized a novel FXR agonist, **FLG390**, and compared its biological effects in osteoblast differentiation with a known FXR agonist, chenodeoxycholic acid (CDCA). As an FXR agonist, **FLG390** accelerated osteoblast differentiation to a comparable extent with CDCA, enhancing alkaline phosphatase (ALP) activity and the expression of osteoblast differentiated-related genes such as ALP, collagen type 1 α1 chain (COL1A1), and runt-related transcription factor 2 (RUNX2). FXR activation elevated the expression of cyclooxygenase (COX)-2 and the production of prostaglandin (PG) E_2_ in the early phase of osteoblast differentiation. A selective COX-2 inhibitor and an antagonist of EP4 receptors, one of PGE_2_ receptors, partially suppressed FXR-activated osteoblast differentiation. Moreover, treatment with either inhibitor during the first 6 h after initiating osteoblast differentiation repressed FXR-activated osteoblast differentiation to the same extent as did the treatment for 6 d. Therefore, a novel FXR agonist, **FLG390,** exhibited potency comparable to CDCA. FXR activation promoted the early phase of osteoblast differentiation via the COX-2-PGE_2_-EP4 axis, representing a potential target for control of bone metabolism.

## 1. Introduction

Bone mass is regulated by the balance between osteoblast-driven bone formation by osteoblasts and bone resorption by osteoclasts [1]. Osteoblasts are derived from mesenchymal stem cells (MSCs) that can differentiate into multiple cell types such as osteoblasts, adipocytes, chondrocytes, and myocytes [2]. Osteoblasts play a critical role in bone formation by producing bone matrix proteins and promoting tissue mineralization [1]. Osteoblast differentiation is controlled by a variety of factors such as bone morphogenetic proteins (BMPs), Wnt, SMADs, β-catenin, runt-related transcription factor 2 (Runx2, also known as core binding factor α1: Cbfa1), and activation of transcription factor (ATF) 4 [3]. Among them, the most important regulators are BMPs that are members of the transforming growth factor (TGF)-β superfamily [4,5].

Farnesoid X receptor (FXR: NR1H4) is a member of the nuclear receptor superfamily that activates gene expression in a ligand-dependent manner [6,7]. Bile acids are the physiological ligands of FXR and enhance the transcription of the FXR target genes [8,9]. Chenodeoxycholic acid (CDCA) is a hydrophobic bile acid that serves as an endogenous FXR ligand [10]. In the FXR-activated gene expression, FXR forms a heterodimer with retinoid X receptors (RXRs), and the FXR-RXR heterodimer binds to an inverted repeat-1 (IR-1) response element in the promoter of the FXR target genes [7]. FXR regulates various metabolic pathways such as bile acid, glucose, and lipid metabolism in various organs [7]. In the bone metabolism, bile acids activate the differentiation of MSCs into osteoblasts via FXR [11]. Moreover, the FXR agonists activate osteoblast differentiation in mouse bone marrow-derived MSCs [12]. Ablation of the FXR gene lowers bone metabolism [13]. Thus, the development of FXR modulators holds great promise in the clinical setting.

Prostaglandins (PGs) have numerous physiological functions, including the regulation of bone metabolism [14]. PGE_2_ activates osteoblast differentiation in bone marrow stromal cells (BMSCs) and calvaria osteoblasts [15,16]. PGF_2α_ is involved in the regulation of bone mineralization [17]. 15-deoxy-Δ^12,14^-PGJ_2_ and Δ^12^-PGJ_2_, which are naturally occurring PGD_2_ derivatives, promote bone metabolism [18,19]. The prostacyclin receptor agonist enhances osteoblast differentiation and increases bone mass in mice [20]. Moreover, cyclooxygenases-2 (COX-2) plays a key role in the production of PGE_2_ in osteoblasts [21]. Non-steroidal anti-inflammatory drugs (NSAIDs) regulate the maturation of matrix and osteoblast differentiation [22,23]. In COX-2 gene knockout (KO) mice, BMSCs exhibit impaired osteoblast differentiation [24]. In addition, overexpression of FXR increases COX-2 expression in chronic obstructive pulmonary disease [25]. However, the molecular mechanism of FXR-activated osteoblast differentiation remains unclear. In the present study, we synthesized and validated a novel FXR agonist and investigated the molecular mechanism underlying the FXR-mediated activation of osteoblast differentiation in mouse ST-2 MSCs.

## 2. Results

### 2.1. Synthesis of FXR Agonist and Validation of Binding Ability and Specificity to FXR

**Scheme 1:**
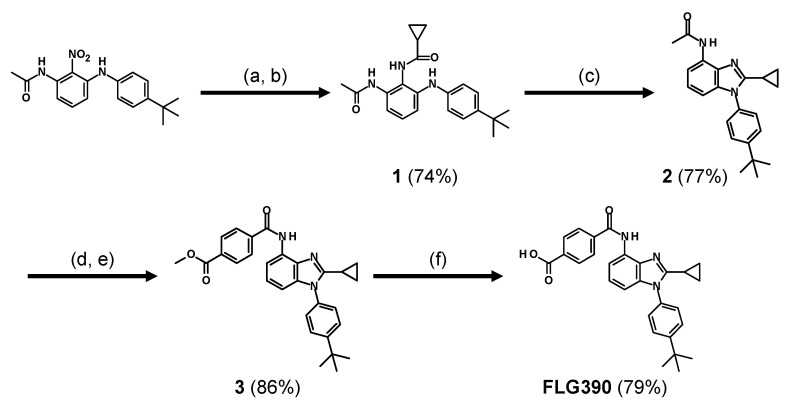
Reagents and conditions: (**a**) 10% Pd/C, H_2_, and methanol at room temperature for 1 h; (**b**) cyclopropanecarboxylic acid, 1-(3-dimethylaminopropyl)-3-ethylcarbodiimide hydrochloride (WSCI-HCl), HOAt (^3^H-[1,2,3]triazolo [4,5-b]pyridin-3-ol), and *N*,*N*-dimethylformamide (DMF) at room temperature for 15 h; (**c**) CH_3_COOH at 80 °C for 4 h; (**d**) 6 M HCl and dioxane at 90 °C for 1 h; (**e**) monomethyl terephthalate, HOAt, WSCI-HCl, and DMF at room temperature for 15 h; (**f**) 1 M NaOH and tetrahydrofuran (THF)/methanol at room temperature for 15 h.

**Figure 1:**
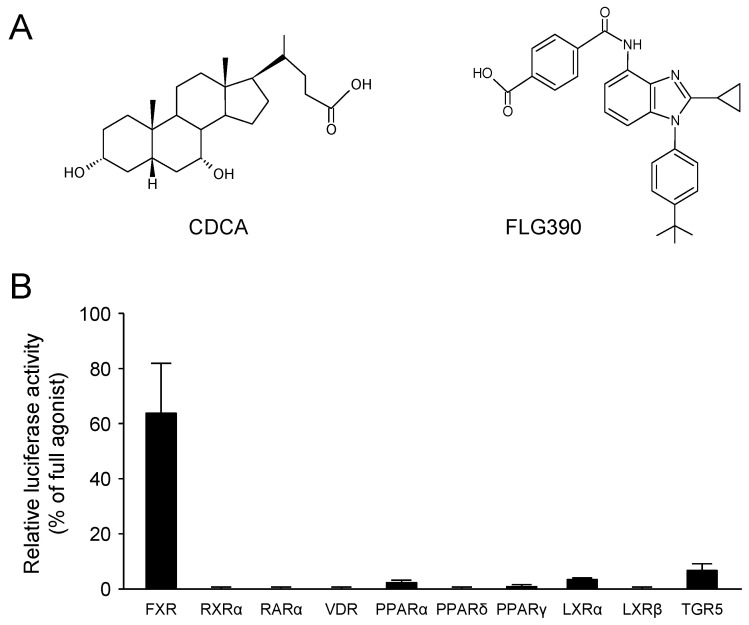
Selectivity screening of **FLG390** against human nuclear receptors and TGR5. (**A**) Structure of FXR agonists. (**B**) Specificity of **FLG390** to nuclear receptors. The agonist activity of **FLG390** for each nuclear receptor was determined at a concentration of 1 μM in the luciferase reporter assay. The vertical axis shows the relative luciferase activity to the maximum activity of the following full agonists for each receptor: FXR—GW4064; RXRα—9-*cis*-retinoic acid; RARα—all-*trans* retinoic acid; VDR—1α,25-dihydroxyvitamin D_3_; PPARα—GW7647; PPARδ—GW501516; PPARγ—GW1929; LXRα and LXRβ—T0901317; and TGR5—lithocholic acid.

FXR is implicated in various disorders [26], suggesting that it holds potential as a therapeutic target. Various FXR modulators have been developed [27]. We previously synthesized FXR modulators and validated their biological properties [12,28,29,30,31]. Herein, we synthesized a novel FXR agonist, **FLG390** (Scheme 1, Figure 1A).

Luciferase reporter assay demonstrated that **FLG390** showed no affinity (less than 5%) to any members of the nuclear receptor superfamily other than FXR, including retinoid X receptor (RXR) α, retinoic acid receptor (RAR) α, vitamin D receptor (VDR), peroxisome proliferator-activated receptor (PPAR) α, δ, and γ, liver X receptor (LXR) α and β, nor to transmembrane G protein-coupled receptor 5 (TGR5), indicating that **FLG390** is a selective FXR agonist (Figure 1B). The binding ability of **FLG390** was validated by a time-resolved fluorescence resonance energy transfer (TR-FRET) assay. The potencies of **FLG390** and CDCA were EC_50_ = 0.63 ± 0.26 µM and 4.49 ± 1.49 µM, and their maximum agonistic activities were 25.1 ± 9.5% and 147.3 ± 4.0%, relative to that of GW4064, a synthetic FXR agonist (0.5 μM).

### 2.2. Activation of Osteoblast Differentiation by FXR Agonist in BMP-2-Induced Mouse ST-2 MSCs

**Figure 2:**
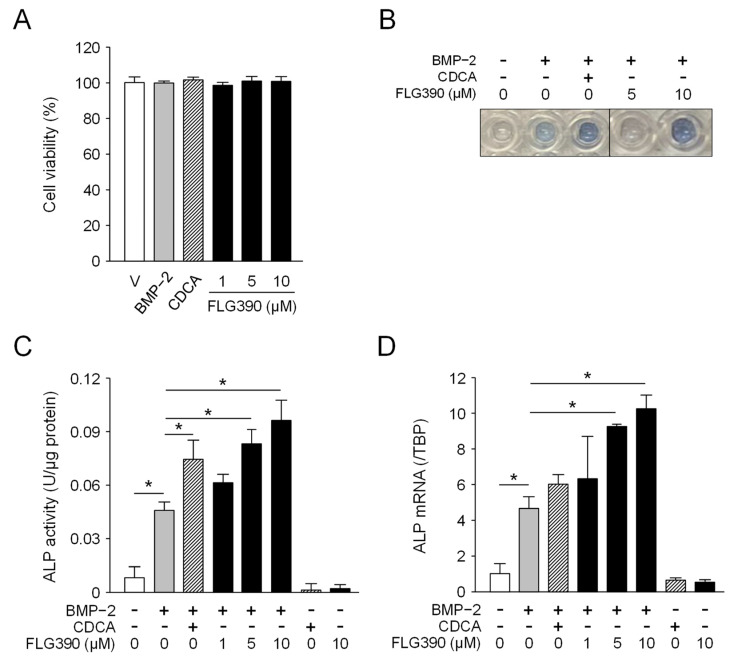
ALP activity and mRNA expression in the FXR agonist-treated ST-2 MSCs. (**A**) Cell viability of ST-2 MSCs. The cells were cultured for 24 h in the medium in the absence (V: vehicle; white column) or presence of BMP-2 (50 ng/mL; gray column) and/or CDCA (10 µM; hatched column) or **FLG390** (1, 5, 10 µM; black columns). The data are presented as the mean ± S.D. (*n* = 8). (**B**) ALP staining. ST-2 MSCs were differentiated into osteoblasts for 6 d in the medium containing BMP-2 (50 ng/mL) with or without CDCA (10 µM) or **FLG390** (5, 10 µM). (**C**) ALP activity in BMP-2- and FXR agonist-treated ST-2 MSCs. The cells were differentiated into osteoblasts for 6 d in the medium with BMP-2 (50 ng/mL; gray column) and/or CDCA (10 µM; hatched column) or **FLG390** (1, 5, 10 µM; black columns). The data are presented as the mean ± S.D. from three independent experiments. * *p* < 0.05. (**D**) Expression of the ALP mRNA in BMP-2- and FXR agonist-treated ST-2 MSCs. The cells were cultured as stated in the legend of Figure 2C. The data are shown as the mean ± S.D. from three independent experiments. * *p* < 0.05.

First, we investigated the cell toxicity of the FXR agonists and BMP-2 in ST-2 MSCs. CDCA and **FLG390** showed no cytotoxicity up to 10 µM (Figure 2A). In addition, BMP-2 (50 ng/mL) was not toxic to ST-2 MSCs.

We measured the activity of ALP that is considered as an initial marker of osteoblast differentiation. ST-2 MSCs were cultured for 6 d in the medium containing BMP-2 and the FXR agonist. ALP staining demonstrated that BMP-2 enhanced ALP activity (Figure 2B and Figure S1), and co-treatment with CDCA or **FLG390** further increased BMP-2-induced ALP activity. The enzymatic activity of ALP was upregulated by treatment with BMP-2 and its activity was further enhanced by co-treatment with CDCA or various concentrations of **FLG390** (1, 5, and 10 μM) (Figure 2C), while treatment with CDCA or **FLG390** alone did not enhance ALP activity (Figure 2C).

Next, we investigated ALP gene expression in BMP-2-induced ST-2 MSCs cultured in the presence of each FXR agonist. The cells were cultured for 6 d in the medium containing CDCA or various concentrations of **FLG390** (1, 5, and 10 μM). BMP-2 elevated the ALP mRNA levels in ST-2 MSCs (Figure 2D). Treatment with BMP-2 and CDCA further upregulated ALP gene expression. **FLG390** also enhanced the expression of the ALP mRNA in BMP-2-induced ST-2 MSCs in a concentration-dependent manner (Figure 2D). Meanwhile, CDCA or **FLG390** alone did not activate the transcription of the ALP gene, in line with our results on ALP activity (Figure 2C). Moreover, the expression of collagen type 1 α1 chain (COL1A1) and runt-related transcription factor 2 (RUNX2), which are the osteogenesis-related genes, was not enhanced by CDCA or **FLG390** alone (Figure S2). Taken together, these results reveal that CDCA and **FLG390** enhanced BMP-2-induced osteoblast differentiation in ST-2 MSCs.

**Figure 3:**
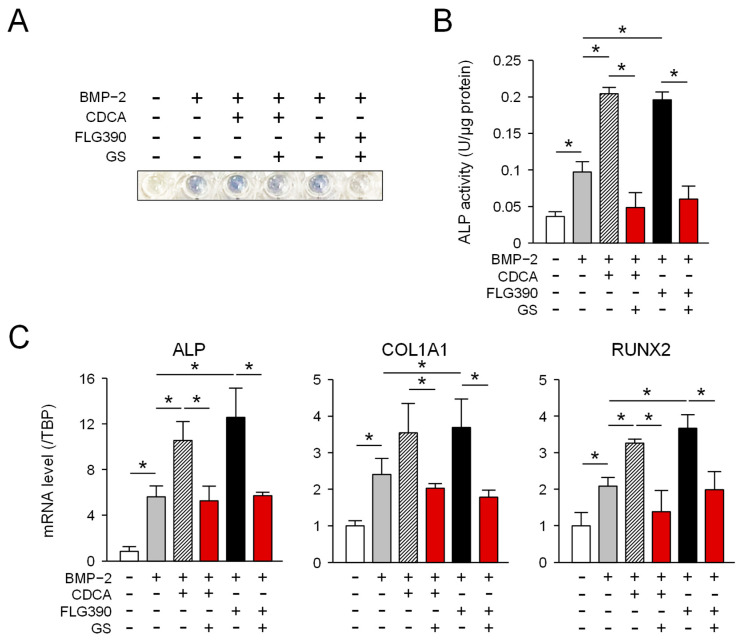
Activation by FXR agonists through FXR in ST-2 MSCs. (**A**) ALP staining. ST-2 MSCs were differentiated into osteoblasts for 6 d in the presence of BMP-2 (50 ng/mL) and/or CDCA (10 µM) or **FLG390** (10 µM) with or without guggulsterone (GS; 25 μM). (**B**) ALP activity. ST-2 MSCs were differentiated into osteoblasts for 6 d in the medium containing BMP-2 (50 ng/mL; gray column) and/or CDCA (10 µM; hatched column) or **FLG390** (10 µM; black columns) with or without guggulsterone (GS; 25 μM; red columns). The data are shown as the mean ± S.D. from three independent experiments. * *p* < 0.05. (**C**) Expression of the osteogenesis-related genes by BMP-2- and FXR agonist-treated ST-2 MSCs. The cells were cultured as described in the legend of Figure 3B. The data are represented as the mean ± S.D. from three independent experiments. * *p* < 0.05.

We examined whether FXR agonist-activated osteoblast differentiation occurs through FXR in ST-2 MSCs. CDCA- or **FLG390**-activated ALP activity was clearly suppressed by co-treatment with guggulsterone, an FXR antagonist, in BMP-2-induced ST-2 MSCs (Figure 3A,B). We also measured the mRNA levels of the osteogenesis-related genes, such as ALP, COL1A1, and RUNX2 in BMP-2- and FXR agonist-treated ST-2 MSCs. The expression levels of these osteogenesis-related genes were elevated by treatment with BMP-2 and further enhanced by co-treatment with CDCA or **FLG390** (Figure 3C). Moreover, the FXR agonist-enhanced expression of these genes was repressed by co-treatment with guggulsterone. These results indicate that CDCA and **FLG390** enhanced the expression of the osteogenesis-related genes and ALP activity via FXR in BMP-2-induced ST-2 MSCs.

### 2.3. Expression of FXR and Osteogenesis-Related Genes During Osteoblast Differentiation in BMP-2/FXR Agonist-Treated Mouse ST-2 MSCs

**Figure 4:**
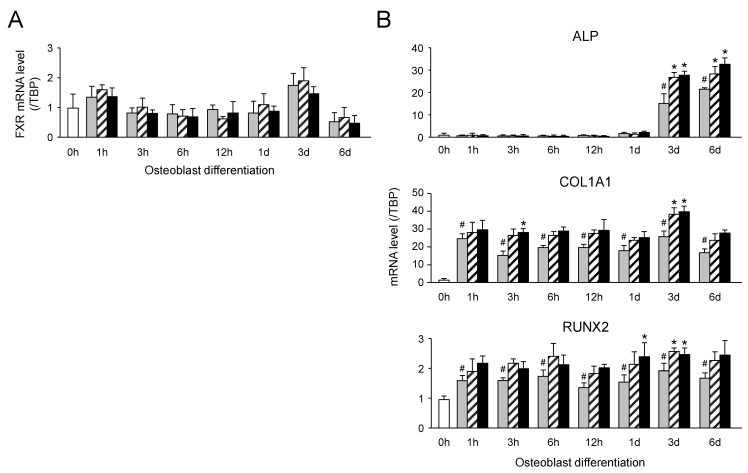
Expression of FXR and osteogenesis-related genes during osteoblast differentiation in BMP-2-induced ST-2 MSCs. (**A**) Expression of the FXR gene during osteoblast differentiation of BMP-2-induced ST-2 MSCs in the presence or absence of the FXR agonist. The cells were differentiated into osteoblasts for 6 d in the medium containing BMP-2 (50 ng/mL; gray columns), and/or CDCA (10 µM; hatched columns) or **FLG390** (10 µM; black columns). The data are shown as the mean ± S.D. from three independent experiments. (**B**) Expression of the osteogenesis-related genes during osteoblast differentiation of BMP-2-induced ST-2 MSCs in the presence or absence of the FXR agonist. The cells were cultured as described in the legend of Figure 4A. The data are represented as the mean ± S.D. from three independent experiments. # *p* < 0.05, vs. 0 h; * *p* < 0.05, vs. BMP-2 alone-treated cells at each time point.

We investigated the expression of the FXR and osteogenesis-related genes such as ALP, COL1A1, and RUNX2 in the presence of BMP-2 and/or the FXR agonist during the osteoblast differentiation of ST-2 MSCs. FXR was consistently expressed during BMP-2-induced osteoblast differentiation without significant changes even in the presence of each of the FXR agonists (Figure 4A). The expression of the ALP gene was low until 1 d after the initiation of treatment with BMP-2 and was then induced during the 6-day osteoblast differentiation (Figure 4B). The FXR agonists further enhanced ALP gene expression, as compared with BMP-2-induced cells. The mRNA expression of the COL1A1 and RUNX2 genes was induced at 1 h after the initiation of BMP-2 treatment and was further enhanced by co-treatment with each FXR agonist (Figure 4B). These results reveal that the FXR agonists, CDCA and **FLG390**, enhanced the expression of the osteogenesis-related genes in BMP-2-induced ST-2 MSCs. Moreover, **FLG390** showed comparable potency as the FXR agonist to CDCA.

### 2.4. Expression of PGE_2_ Synthase and Production of PGE_2_ During Osteoblast Differentiation of BMP-2/FXR Agonist-Treated Mouse ST-2 MSCs

**Figure 5:**
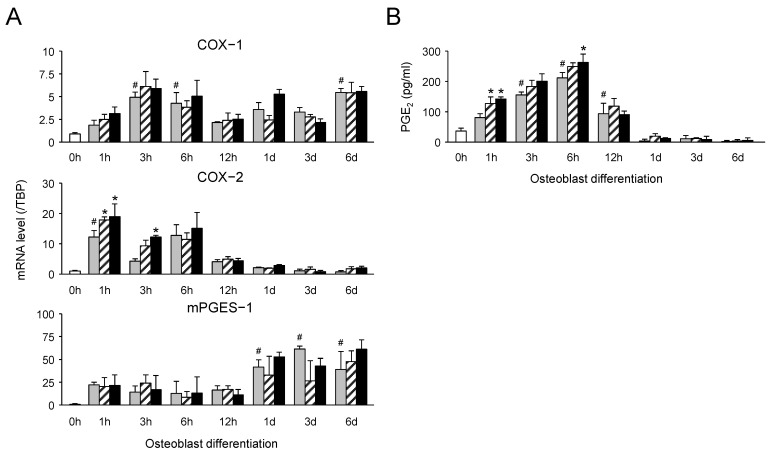
Expression of PGE_2_ synthases in BMP-2-induced ST-2 MSCs. (**A**) Expression levels of the COX-1, COX-2, and mPGES-1 genes during osteoblast differentiation of BMP-2-induced ST-2 MSCs in the presence or absence of the FXR agonist. The cells were differentiated into osteoblasts for 6 d in the medium containing BMP-2 (50 ng/mL; gray columns) with or without CDCA (10 µM; hatched columns) or **FLG390** (10 µM; black columns). The data are presented as the mean ± S.D. from three independent experiments. # *p* < 0.05, vs. 0 h; * *p* < 0.05, vs. BMP-2 alone-treated cells at each time point. (**B**) PGE_2_ production during the osteoblast differentiation of BMP-2-induced ST-2 MSCs in the presence of the FXR agonist. The cells were cultured as stated in the legend of Figure 5A and further incubated with A23187 (5 µM) for 15 min at 37 °C. The data are shown as the mean ± S.D. from three independent experiments. # *p* < 0.05, vs. 0 h; * *p* < 0.05, vs. BMP-2 alone-treated cells at each time point.

PGs are associated with the regulation of bone metabolism. PGE_2_ activates bone formation [14,32,33]. COX-2 KO mice exhibited impaired osteoblast differentiation of BMSCs [24]. Bile acid, as an endogenous FXR agonist, induces COX-2 expression in human gastric cancer cells [34]. We investigated the involvement of COX expression and PGE_2_ production in the regulation of FXR-mediated activation of osteoblast differentiation. COX-1 was consistently expressed during osteoblast differentiation with a slight elevation by BMP-2 treatment, and these expression levels were not significantly altered when the cells were co-treated with each of the FXR agonists (Figure 5A). Meanwhile, COX-2 expression was elevated by treatment with BMP-2 in the early phase (1–6 h after the treatment) of osteoblast differentiation and then was lowered to basal levels (Figure 5A). Moreover, the COX-2 mRNA levels were further elevated by co-treatment with each FXR agonist when compared with BMP-2 alone (Figure 5A). On the other hand, CDCA and **FLG390** alone did not enhance COX-2 expression (Figure S3). The microsomal PGE_2_ synthase-1 (mPGES-1) mRNA levels increased at 1 h and maintained until 12 h after the initiation of BMP-2 treatment, whereafter they elevated (Figure 5A). On the other hand, when the cells were treated with BMP-2 and the FXR agonist, the mPGES-1 mRNA levels were not changed, as compared with BMP-2 alone (Figure 5A). Next, we measured the PGE_2_ levels during osteoblast differentiation. PGE_2_ was produced in ST-2 MSCs, and its production peaked at 6 h after the initiation of BMP-2-induced osteoblast differentiation, followed by a gradual decrease to basal levels (Figure 5B). Furthermore, PGE_2_ production was further increased by co-treatment with CDCA or **FLG390**, and continued to increase until 6 h after the initiation of osteoblast differentiation, followed by a gradual decrease to basal levels (Figure 5B). The pattern of BMP-2- and FXR agonist-mediated PGE_2_ production during osteoblast differentiation resembled that of COX-2 expression (Figure 5A). These results suggest that COX-2 expression and PGE_2_ production increased at 1 h after treatment with BMP-2 and were maintained for up to 6 h, an effect that was elevated by treatment with the FXR agonist in ST-2 MSCs.

### 2.5. Suppression of FXR Agonist-Activated Osteoblast Differentiation by COX Inhibitor in BMP-2/FXR Agonist-Treated Mouse ST-2 MSCs

**Figure 6:**
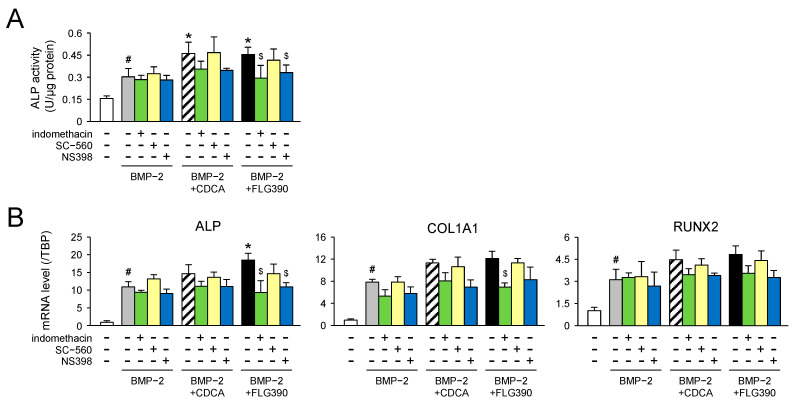
Change in expression of osteogenesis-related genes by COX inhibitors in BMP-2-induced ST-2 MSCs. (**A**) ALP activity. ST2-MSCs were differentiated into osteoblasts for 6 d in the medium containing BMP-2 (50 ng/mL; gray columns) with or without CDCA (10 µM; hatched columns) or **FLG390** (10 µM; black columns) in the presence of each of indomethacin (1 µM; green columns), SC-560 (100 nM; yellow columns), and NS398 (1 µM; blue columns). The cells were pre-incubated with each COX inhibitor for 30 min before initiation of osteoblast differentiation. The data are shown as the mean ± S.D. from three independent experiments. # *p* < 0.05, vs. vehicle; * *p* < 0.05, vs. BMP-2 alone-treated cells in each treatment. ^$^ *p* < 0.05, vs. BMP-2- and **FLG390**-treated cells. (**B**) Expression of the osteogenesis-related genes. ST-2 MSCs were cultured as stated in the legend of Figure 6A. The data are presented as the mean ± S.D. from three independent experiments. # *p* < 0.05, vs. vehicle; * *p* < 0.05, vs. BMP-2 alone-treated cells in each treatment. ^$^ *p* < 0.05, vs. BMP-2- and **FLG390**-treated cells.

We examined the involvement of COX-2 in regulating FXR agonist-activated osteoblast differentiation in BMP-2-induced ST-2 MSCs. The cells were differentiated into osteoblasts in the presence of a non-selective COX-1/2 inhibitor, indomethacin, a selective COX-1 inhibitor, SC-560, and a selective COX-2 inhibitor, NS398. FXR agonist-induced ALP activity was partially suppressed by indomethacin or NS398, but not by SC-560, in BMP-2-induced ST-2 MSCs (Figure 6A). Treatment with indomethacin or NS398, but not with SC-560, partially lowered the expression of ALP, COL1A1, and RUNX2 in BMP-2- and FXR agonist-treated ST-2 MSCs (Figure 6B). These results reveal that the FXR agonist activated osteoblast differentiation by enhancing COX-2 expression in BMP-2-induced ST-2 MSCs.

### 2.6. Suppression of FXR Agonist-Activated Osteoblast Differentiation by EP4 Receptor Antagonist in BMP-2/FXR Agonist-Treated Mouse ST-2 MSCs

**Figure 7:**
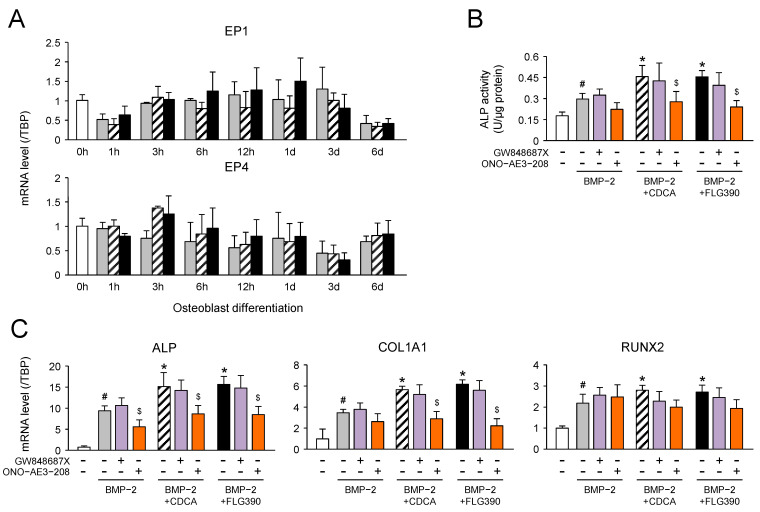
Expression of EP receptors during osteoblast differentiation in BMP-2-induced ST-2 MSCs. (**A**) Expression of the EP1 and EP4 receptor genes during osteoblast differentiation in BMP-2-induced ST-2 MSCs. The cells were differentiated into osteoblasts for 6 d in the medium containing BMP-2 (50 ng/mL; gray columns) with or without CDCA (10 µM; hatched columns) or **FLG390** (10 µM; black columns). The data are presented as the mean ± S.D. from three independent experiments. # *p* < 0.05, vs. 0 h; * *p* < 0.05, vs. BMP-2 alone-treated cells. (**B**) ALP activity. ST2-MSCs were differentiated into osteoblasts for 6 d in the medium containing BMP-2 (50 ng/mL; gray columns) with or without CDCA (10 µM; hatched columns) or **FLG390** (10 µM; black columns) in the presence or absence of GW848687X, an EP1 receptor antagonist (20 nM; purple columns) or ONO-AE3-208, an EP4 receptor antagonist (10 nM; orange columns). The cells were pre-incubated with each EP receptor antagonist for 30 min before the initiation of osteoblast differentiation. The data are shown as the mean ± S.D. from three independent experiments. # *p* < 0.05, vs. 0 h; * *p* < 0.05, vs. BMP-2 alone-treated cells; ^$^ *p* < 0.05, vs. BMP-2- and CDCA- or **FLG390**-treated cells in each treatment. (**C**) Expression level of the osteogenesis-related genes in the EP1 or EP4 receptor antagonist-treated BMP-2-induced ST-2 MSCs. The cells were cultured as described in the legend of Figure 7B. The data are shown as the mean ± S.D. from three independent experiments. # *p* < 0.05, vs. 0 h; * *p* < 0.05, vs. BMP-2 alone-treated cells; ^$^ *p* < 0.05, vs. BMP-2- and CDCA- or **FLG390**-treated cells in each treatment.

PGE_2_ shows the physiological properties by binding to four PGE_2_ receptor subtypes; EP1, EP2, EP3, and EP4 [35]. To further examine the FXR agonist-mediated enhancement of osteoblast differentiation, we measured the expression of the EP receptor genes in BMP-2-induced ST-2 MSCs. The EP1 and EP4 mRNAs were consistently expressed during osteoblast differentiation with slight changes following BMP-2 treatment (Figure 7A). In contrast, the expression of EP2 and EP3 receptors was under the detection limit in our experimental conditions.

Next, we examined whether EP1 and EP4 receptors are involved in the regulation of FXR agonist-activated osteoblast differentiation in BMP-2-induced ST-2 MSCs. The cells were incubated for 6 d in the medium in the presence of BMP-2 and an EP1 receptor antagonist, GW848687X, or an EP4 receptor antagonist, ONO-AE3-208. ALP activity was lowered by treatment with ONO-AE3-208 in BMP-2- and FXR agonist-treated ST-2 MSCs (Figure 7B). Moreover, when the cells were incubated with ONO-AE3-208, the FXR agonist-mediated increases in the ALP, COL1A1, and RUNX2 mRNA expression were repressed in BMP-2-induced ST-2 MSCs (Figure 7C). In contrast, the EP1 receptor antagonist had no effect on their expression (Figure 7B,C). These results suggest that FXR agonist-activated osteoblast differentiation was mediated via EP4 receptors in BMP-2-induced ST-2 MSCs. Furthermore, EP4 receptor expression was not altered by CDCA or FLG390 with or without BMP-2 (Figure 7A and Figure S3).

### 2.7. Activation of Early Phase of Osteoblast Differentiation by COX-2-Mediated PGE_2_ in BMP-2/FXR Agonist-Treated Mouse ST-2 MSCs

**Figure 8:**
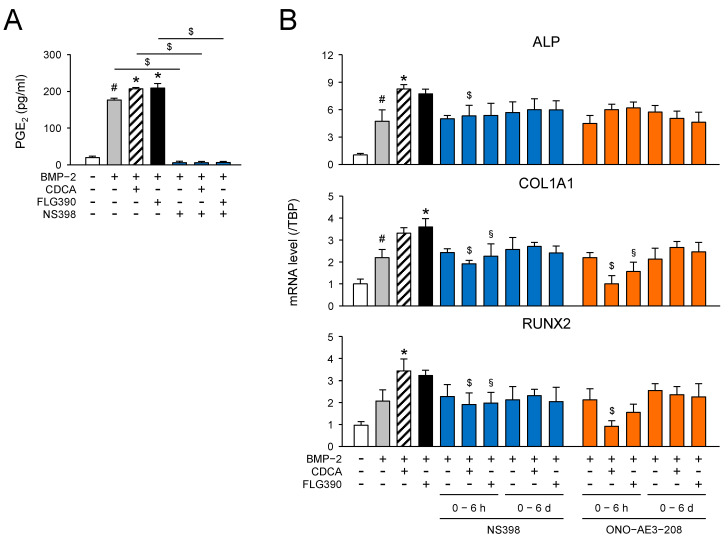
Phase-specific effect of inhibition of COX-2 or EP4 receptors during osteoblast differentiation in BMP-2- and FXR agonist-induced ST-2 MSCs. (**A**) Inhibition of PGE_2_ production by NS398 in the presence or absence of BMP-2 and/or CDCA or **FLG390** in ST-2 MSCs. The cells were incubated for 6 h in the medium containing BMP-2 (50 ng/mL) with or without CDCA (10 µM), **FLG390** (10 µM), and NS398 (1 µM), and further incubated with A23187 (5 µM) for 15 min at 37 °C. # *p* < 0.05, vs. 0 h; * *p* < 0.05, vs. BMP-2 alone-treated cells; ^$^ *p* < 0.05. (**B**) Phase-specific effect of COX-2 inhibitor or EP4 receptor antagonist during the osteoblast differentiation of BMP-2- and FXR agonist-treated ST-2 MSCs. The cells (white columns) were differentiated into osteoblasts for 6 d in the medium containing BMP-2 (50 ng/mL; gray columns) with or without CDCA (10 μM) or **FLG390** (10 μM; black columns) in the presence or absence of NS398 (1 µM) or ONO-AE3-208 (10 nM) during 0–6 h or 0–6 d of 6-day osteoblast differentiation. The cells were pre-incubated with NS398 or ONO-AE3-208 for 30 min prior to the initiation of osteoblast differentiation. The data are shown as the mean ± S.D. from three independent experiments. # *p* < 0.05, vs. 0 h; * *p* < 0.05, vs. BMP-2 alone-treated cells; ^$^ *p* < 0.05, vs. BMP-2- and CDCA-treated cells, ^§^ *p* < 0.05, vs. BMP-2- and **FLG390**-treated cells.

COX-2-mediated PGE_2_ production increased by treatment with the FXR agonists in the early phase (1–6 h) of osteoblast differentiation (Figure 5B). In addition, FXR agonist-activated osteoblast differentiation was repressed by an EP4 antagonist (Figure 7B,C). We further analyzed the phase-specific effect of FXR agonist-activated osteoblast differentiation in BMP-2-induced ST-2 MSCs. BMP-2- and/or FXR agonist-treated PGE_2_ production was completely inhibited by treatment with NS398 (Figure 8A).

Then, we investigated the effect of the FXR agonist in the presence of NS398 or ONO-AE3-208 on different phases, from 0 to 6 h (0–6 h) and from 0 to 6 d (0–6 d) after the initiation of osteoblast differentiation (Figure 8B). When NS398 or ONO-AE3-208 was present during 0–6 h or 0–6 d of 6-day osteoblast differentiation in BMP-2- and FXR agonist-treated ST-2 MSCs, the BMP-2- and/or FXR agonist-induced expression of osteogenesis-related genes all decreased (Figure 8B). These results reveal that the FXR agonist activated the early phase of osteoblast differentiation through EP4 receptors by enhancing COX-2-mediated PGE_2_ production in BMP-2-induced ST-2 MSCs.

## 3. Discussion

FXR plays an important role in various metabolic pathways including glucose and lipid metabolisms [7]. The effects of FXR modulators in the metabolic diseases have been extensively studied [36]. We previously synthesized the FXR modulators and investigated their biological activities [12,28,29,30,31]. During the development of the FXR modulators, one of the 1,4-disubstituted benzimidazole derivatives served as an FXR/PPARγ dual partial agonist [31]. As the 1,2,4-trisubstituted benzimidazole analog, **FLG390** was a selective and partial FXR agonist, and its effect on osteoblast differentiation was investigated in mouse ST-2 MSCs. **FLG390** exhibited comparable potency as the FXR agonist to CDCA, a known FXR agonist, in the BMP-2-induced osteoblast differentiation of ST-2 MSCs.

Bone remodeling consists of two important processes, bone formation and bone resorption, driven by two cell lineages, namely, osteoblasts and osteoclasts [37]. Osteoblasts activate bone growth and are derived from MSCs that are stem cells having the abilities to differentiate into various cell types such as osteoblasts, chondrocytes, myocytes, and adipocytes [38]. Osteoblast differentiation is regulated by various factors including cytokines and transcription factors [39]. BMP-2 is a member of the TGF-β superfamily that is known to enhance osteoblast differentiation in various MSCs [40]. Nuclear receptors are the ligand-dependent transcription factors, some of which are involved in the regulation of osteoblast differentiation via controlling the expression of the osteogenesis-related genes [41]. FXR is a nuclear receptor and regulates various metabolic pathways including bone metabolism. FXR activation induces osteoblast differentiation of human osteosarcoma SaOS2 cells [11]. Bile acid, an endogenous FXR ligand, promotes osteoblast differentiation [13]. Ablation of FXR decreases bone mass [13], and the deficiency of small heterodimer partner (SHP), an FXR target gene, lowers osteoblast differentiation in primary osteoblasts [42]. Therefore, FXR is a critical transcription factor involved in the regulation of bone metabolism and has a potential for the development of bone diseases. Previously, we reported that FXR activation induces osteoblast differentiation in ST-2 MSCs [12]. In the present study, we synthesized the novel FXR agonist and investigated the activation mechanism of osteoblast differentiation in BMP-2-induced ST-2 MSCs. CDCA and a novel FXR agonist **FLG390** exhibited almost the same potency on the elevated expression of the osteogenesis-related genes and the increased ALP activity in BMP-2-induced ST-2 MSCs (Figure 3B).

PGs are lipid mediators and play important roles in the regulation of a variety of physiological functions, including bone formation, through their specific receptors in an autocrine or paracrine manner [14]. PGE_2_ is the most abundant PG in the body and is known to activate bone formation [33]. COX-2 is expressed in human MSCs, and its inhibition completely suppresses PGE_2_ production [43]. Moreover, suppression of PGE_2_ synthesis by NSAIDs inhibits osteoblast differentiation in rat osteoblasts [44] and human MSCs [45]. Meanwhile, PGE_2_ induces bone formation in BMSCs and calvaria osteoblasts [15,46,47,48]. Administration of PGE_2_ stimulates bone formation in rats and activates osteoblast differentiation in bone marrow precursor cells [49,50]. Delayed bone formation in COX-2 KO mice is rescued by PGE_2_ [24]. Thus, PGE_2_ plays an important role in the regulation of osteoblast differentiation. PGE_2_ exerts its physiological effects by binding to four EP receptors: EP1–EP4 [35]. The role of each EP receptor in the regulation of bone metabolism appears to be cell type-dependent. EP1 receptors are involved in the regulation of osteoblast differentiation of mouse MSCs [51]. On the other hand, EP4 receptors are the major EP receptors in human bone tissues [52], mouse osteoblastic cell line MC3T3-E1 cells, BMSCs [52,53,54], rat calvaria osteoblasts, and human osteoblasts [55]. An EP4 agonist, ONO-4819, enhances bone formation in ovariectomized rats [56,57]. In addition, PGE_2_-activated bone formation is suppressed by an EP4 receptor antagonist, L-161982, in rat BMSCs [58]. An EP4 receptor agonist, ONO-4819, enhances the osteoblast differentiation of various MSCs [59,60]. FXR activation enhanced the early phase of osteoblast differentiation via COX-2-mediated PGE_2_ production through EP4 receptors in BMP-2-induced ST-2 MSCs (Figure 7). However, the COX-2 inhibitor and EP4 receptor antagonist partially suppressed FXR-activated osteoblast differentiation, suggesting the involvement of another mechanism in this regulation. A more precise mechanism of this regulation should be further investigated.

## 4. Materials and Methods

### 4.1. Materials

Chenodeoxycholic acid (CDCA), lithocholic acid, indomethacin, and Fast Blue BB *Salt* were purchased from Sigma-Aldrich (St. Louis, MO, USA). GW4064, (Z)-guggulsterone, T0901317, 1α,25-dihydroxyvitamin D_3_, GW7647, GW1929, SC-560, NS398, GW848687X, and ONO-AE3-208 were obtained from Cayman Chemical (Ann Arbor, MI, USA). GW501516 was purchased from ChemScene LLC (Newark, NJ, USA). All-*trans*-retinoic acid and 9-*cis*-retinoic acid were purchased from FUJIFILM Wako Pure Chemical (Osaka, Japan). Recombinant human BMP-2 was purchased from Miltenyi Biotec (Bergisch Gladbach, Germany). A23187 was obtained from Calbiochem (Merck Millipore, Burlington, MA, USA). Naphthol AS-MX phosphate was purchased from Nacalai Tesque (Kyoto, Japan). Compound characterization and purity determined by RP-HPLC were described in the Supplementary Materials.

### 4.2. Coactivator Recruitment Assay

The binding ability of the FXR agonists was examined using a LanthaScreen^TM^ TR-FRET FXR Coactivator Assay Kit (Thermo Fisher Scientific, Waltham, MA, USA) as reported previously [28]. In brief, various concentrations of compounds (4 × 10^−9^–4 × 10^−5^ M) were incubated with fluorecein-SRC2-2 coactivator peptide, FXR-LBD-Glutathione S-transferase fusion protein, and Lantha-screen Tb-anti GST antibody in a 384-well black plate. The TR-FRET signals were measured by an EnVision Multilabel Reader (PerkinElmer, Waltham, MA, USA). The EC_50_ value was determined by constructing a dose–response curve. The activity for the FXR agonist to GW4064 (2 μM) was shown as the relative agonist activity.

### 4.3. Luciferase Reporter Assay

The luciferase reporter assay was carried out as described previously [29]. Briefly, luciferase activity was measured in the cells transfected with each receptor expression vector and luciferase reporter vector carrying the corresponding response element and agonist. The following are the full agonists and their concentrations for each receptor: GW4064 (100 nM for FXR), 9-*cis*-retinoic acid (100 nM for RXRα), all-*trans* retinoic acid (100 nM RARα), 1α,25-dihydroxyvitamin D_3_ (100 nM for VDR), GW7647 (100 nM for PPARα), GW501516 (100 nM for PPARδ), GW1929 (100 nM for PPARγ), T0901317 (100 nM for LXRα and LXRβ), and lithocholic acid (200 nM for TGR5). The relative activity to the full agonist for each receptor was determined.

### 4.4. Cell Culture

Mouse ST2 MSCs were obtained from RIKEN BioResource Research Center (Tsukuba, Ibaraki, Japan) and were grown in RPMI-1640 (Sigma-Aldrich) supplemented with 10% (*v*/*v*) fetal calf serum (Thermo Fisher Scientific) and antibiotics (100 unit/mL penicillin and 100 μg/mL streptomycin; Nacalai Tesque) at 37 °C under a humidified atmosphere of 5% CO_2_. To induce osteoblast differentiation, ST-2 MSCs were cultured for 6 d in RPMI-1640 supplemented with BMP-2 (50 ng/mL). On day 3, the medium was replaced with fresh RPMI-1640 containing BMP-2. The chemicals were added when the medium was changed. A COX inhibitor or EP receptor antagonist was added to the medium 30 min prior to the initiation of induction with BMP-2.

### 4.5. Cell Viability Assay

ST-2 MSCs were grown in RPMI-1640 supplemented with BMP-2 (50 ng/mL) with or without CDCA (10 µM) or various concentrations of **FLG390** (1, 5, and 10 μM). After 24 h, a Cell Counting Kit-8 (DOJINDO LABORATORIES, Kumamoto, Japan) was employed to measure cell viability according to the manufacturer’s instructions.

### 4.6. RNA Analysis

RNA extraction and first-strand cDNA synthesis were performed as described previously [12]. Quantitative PCR analysis was carried out using a TB Green Fast qPCR Mix (Takara-Bio) on a LightCycler 480 System (Roche Diagnostics, Mannheim, Germany). The primers used in this study are shown in Table 1. The mRNA level of the desired gene was normalized to that of TATA-binding protein (TBP) as an internal control. The relative mRNA levels were analyzed by the 2^−ΔΔCt^ method [61].

### 4.7. ALP Staining

The cells were fixed with 10% (*v*/*v*) formaldehyde in PBS at 25 °C for 10 min. The cells were washed with PBS and incubated at 37 °C for 30 min in 0.1 M Tris-Cl (pH 8.8) containing 50 μM MgCl_2_, 0.6 mg/mL Fast Blue BB salt and 0.1 mg/mL Naphthol AS-MX phosphate. After washing the cells with PBS, the stained cells were analyzed by a microscope (CKX-41, Olympus, Tokyo, Japan).

### 4.8. ALP Activity

ALP activity was measured as described previously [12]. Briefly, the cells were solubilized in PBS containing 1% (*v*/*v*) Nonidet P-40. After two freeze–thaw cycles and centrifugation (1500× *g* for 5 min), the supernatants were used for the measurement of ALP activity by a LabAssay ALP (FUJIFILM WAKO Pure Chemical) according to the manufacturer’s instructions. Protein concentrations were measured by a Protein Assay BCA Kit (Nacalai Tesque). ALP activity was shown by normalizing to protein concentration.x

### 4.9. Measurement of PGE_2_

ST-2 MSCs were incubated in RPMI-1640 with 5 μM A23187 at 37 °C for 15 min. After centrifugation of the medium (700× *g* for 3 min), the supernatants were used to measure the PGE_2_ levels by a Prostaglandin E_2_ ELISA Kit-Monoclonal and Prostaglandin E_2_ Express ELISA Kit (Cayman Chemical) according to the manufacturer’s protocols.

### 4.10. Statistical Analysis

Results were expressed as the means ± S.D. One-way analysis of variance (ANOVA) followed by the Tukey–Kramer post hoc test was performed to assess the statistical significance analysis of multiple comparisons (Statcel 5, OMS Ltd., Tokyo, Japan). A value of *p* < 0.05 was considered statistically significant.

## 5. Conclusions

A novel FXR agonist, **FLG390**, activated osteoblast differentiation with comparable potency to CDCA in BMP-2-induced mouse ST-2 MSCs. FXR activation accelerated the early phase of osteoblast differentiation through EP4 receptors by enhancing COX-2 expression PGE_2_ production in BMP-2-induced ST-2 MSCs. As it has been reported that COX-2 expression and PGE_2_ production are increased in the early phase after fractured tibia [62], the agonists of FXR and EP4 receptors may have potential for the acceleration of the early phase of bone formation after injury. In further studies, in addition to in vivo validation, a more precise regulatory mechanism underlying FXR agonist-activated osteoblast differentiation should be addressed to understand the whole mechanism.

## Figures and Tables

**Table 1:** Nucleotide sequences of primers used in qPCR.

Gene	Accession No.	Forward Primer	Reverse Primer
ALP	NM_007431	5′-AACCCAGACACAAGCATTCC-3′	5′-GAGAGCGAAGGGTCAGTCAG-3′
COL1A1	NM_007742	5′-CATGTTCAGCTTTGTGGACCT-3′	5′-GCAGCTGACTTCAGGGATGT-3′
RUNX2	NM_009820	5′-GCACCGACAGTCCCAACT-3′	5′-CTCCGAGGGCTACAACCTT -3′
FXR	NM_001163700	5′-GGAGAGGAAGACTCAGTCCAGA-3′	5′-GCCTGGACGACAGAGCTAAA-3′
COX-1	NM_008969	5′-CCTCTTTCCAGGAGCTCACA-3′	5′-TCGATGTCACCGTACAGCTC-3′
COX-2	NM_011198	5′-GATGCTCTTCCGAGCTGTG-3′	5′-GGATTGGAACAGCAAGGATTT-3′
mPGES-1	NM_022415	5′-GCACACTGCTGGTCATCAAG-3′	5′-ACGTTTCAGCGCATCCTC-3′
EP1	NM_013641	5′-GAGCCAGGGAGTAGCTGGA-3′	5′-GCTCATATCAGTGGCCAAGAG-3′
EP4	NM_001136079	5′-CCTAACCCCACCCTACAGGT-3′	5′-AGAAGGACGCGTTGACTCC-3′
TBP	NM_013684	5′-GTGATGTGAAGTTCCCCATAAGG-3′	5′-CTACTGAACTGCTGGTGGGTCA-3′

## Data Availability

Data are contained within the article and Supplementary Materials.

## Supplementary Materials

The following supporting information can be downloaded at: https://www.mdpi.com/article/10.3390/molecules30010058/s1, Supplementary Material: Chemistry general; Synthetic protocol for **FLG390**; Figure S1: Uncropped data of ALP staining shown in Figure 2B; Figure S2: Expression of osteogenesis-related gene in the FXR agonist-treated ST-2 MSCs; Figure S3: Expression of osteogenesis-related gene in the FXR agonist-treated ST-2 MSCs.
