# Supplementary material for: FXR Activation Accelerates Early Phase of Osteoblast Differentiation Through COX-2-PGE2-EP4 Axis in BMP-2-Induced Mouse Mesenchymal Stem Cells
Source: Molecules. 2024 Dec 27;30(1):58. doi: 10.3390/molecules30010058 (PMC11722014; doi:10.3390/molecules30010058)
Supplement: Supplementary file 1 [file molecules-30-00058-s001.zip › molecules-3342796-supplementary.pdf]

Supplemental information

Supplemental material

Chemistry general

All chemicals were purchased from Tokyo Chemical Industry (Tokyo, Japan) and FUJIFILM Wako Pure Chemical (Osaka, Japan) and used without further purification. ^{1}H-NMR experiments were recorded on a JMTC-600 (JEOL, Tokyo, Japan) 600 NMR spectrometer with $CDCl_3$ or DMSO-d_6 as solvents. Chemical shifts were expressed in parts per million (ppm, δ) and referred to the solvent signal. HRMS spectra were recorded on the AccuTOF (JMS-T100LC) equipped with an electrospray ion source (JEOL). The analytical HPLC system consisted of a SHINADZU CBM-20A System Controller and a SHIMADZU Pump Unit LC-20AT, a SHIMADZU In-Line Degasser DGU-20A3R, a SHIMADZU SPD-20A Absorbance Detector, a SHIMADZU FCV-11AL Valve Unit and a SHIMADZU FRC-10A Fraction Collector (SHIMADZU, Kyoto, Japan). The absorbance detector was operated at 254 nm. The mobile phase for preparation and analysis was a combination of water (A) and acetonitrile (B), both containing 0.1% TFA, and the flow rate was 1 mL/min. TSK gel ODS column (4.6 x 150 mm, TOSOH, Tokyo, Japan) was used.

*Synthetic protocol for **FLG390***

N-[2-Acetamido-6-(4-tert-butylanilino)phenyl]cyclopropanecarboxamide (1):

N-[3-(4-*tert*-Butylanilino)-2-nitro-phenyl]acetamide (250 mg, 0.76 mmol) [1] in dist.MeOH (15 mL) was hydrogenated over a 10% Pd/C (75 mg) at room temperature for 1 h. The solution was filtered through celite and concentrated to provide a red oily product (N-[2-amino-3-(4-*tert*-butylanilino)phenyl] acetamide). The former product was dissolved in DMF (5 mL), and cyclopropanecarboxylic acid (90 μL, 1.14 mmol), HOAt (224 mg, 1.71 mmol) and WSCI-HCl (330 mg, 1.71 mmol) were added at ambient temperature. The reaction mixture was stirred at ambient temperature for 15 h. The reaction mixture was quenched with saturated $NaHCO_3$ and extracted with ethyl acetate. The combined extracts were washed with H_2O and brine, dried over $MgSO_4$ and concentrated under reduced pressure. The residue was purified by silica gel column chromatography to give **Compound 1** in 74% yield. Rf = 0.45 (*n*-hexane/ethyl acetate = 1/2), ^{1}H NMR (600 MHz, $CDCl_3$): δ 8.18 (s, 1H), 8.10 (s, 1H), 7.27–7.26 (m, 2H), 7.15–7.12 (m, 3H), 6.85 (d, J = 8.6 Hz, 2H), 5.90 (s, 1H), 2.17 (s, 3H), 1.51–1.50 (m, 1H), 1.30 (s, 9H), 1.04–1.02 (m, 2H), 0.83–0.81 (m, 2H).

N-[1-(4-tert-Butylphenyl)-2-cyclopropyl-benzimidazol-4-yl] acetamide (2):

Compound **1** (136 mg, 0.37 mmol) was dissolved in formic acid (9 mL) at ambient temperature and stirred at 80 °C for 4 h. The solvent was evaporated and the residue was adjusted pH to 8–9 with sat.$NaHCO_3$ and extracted with ethyl acetate. The combined extracts were washed with H_2O and brine, dried over $MgSO_4$ and concentrated under reduced pressure. The residue was purified by silica gel column chromatography to give **Compound 2** in 77% yield. Rf = 0.45 (*n*-hexane/ethyl acetate = 1/2), ^{1}H NMR (600 MHz, $CDCl_3$): δ 8.46 (s, 1H), 8.18 (d, J = 7.9 Hz, 1H), 7.57 (d, J = 8.5 Hz, 2H), 7.38 (d, J = 8.5 Hz, 2H), 7.13 (t, J = 8.1 Hz, 1H), 6.87 (d, J = 8.1 Hz, 1H), 2.31 (s, 3H), 1.89–1.87 (m, 1H), 1.41 (s, 9H), 1.27–1.25 (m, 2H), 1.05–1.06 (m, 2H).

Methyl 4-[[1-(4-tert-butylphenyl)-2-cyclopropyl-benzimidazol-4-yl]carbamoyl]benzoate (3):

Compound **2** (97 mg, 0.28 mmol) was dissolved in dioxane (3 mL). To the former solution was added 6 M HCl (0.46 mL, 2.8 mmol) at ambient temperature and the reaction mixture was stirred at 90 ^{o}C for 2 h. The solvent was evaporated and the residue was adjusted pH to 8 with sat.$NaHCO_3$ and extracted with ethyl acetate. The combined extracts were washed with H_2O and brine, dried over $MgSO_4$ and concentrated under reduced pressure to give crude product. Oxalyl chloride (43 μL, 0.5 mmol) and DMF (a few drops) were added in turn to a solution of monomethyl terephthalate (60 mg, 0.34 mmol) in CH_2Cl_2 (3 mL) at ambient temperature and the resultant mixture was stirred at ambient temperature for 1.5 h. The mixture was evaporated, and the residue was dissolved in CH_2Cl_2 (5 mL). The former crude product and triethylamine (105 μL, 0.75 mmol) were added in turn and the reaction mixture was stirred at ambient temperature for 1 h. The reaction mixture was quenched with sat.NH_4Cl and extracted with ethyl acetate. The combined extracts were washed with H_2O and brine, dried over $MgSO_4$ and concentrated under reduced pressure. The residue was purified by silica gel column chromatography to give **Compound 3** in 86% yield. Rf = 0.4 (*n*-hexane/ethyl acetate = 2/1), ^{1}H NMR (600 MHz, $CDCl_3$): δ 9.20 (s, 1H), 8.33 (d, J = 7.9 Hz, 1H), 8.20 (d, J = 8.5 Hz, 2H), 8.08 (d, J = 8.5 Hz, 2H), 7.59 (d, J = 8.5 Hz, 2H), 7.41 (d, J = 8.5 Hz, 2H), 7.20 (t, J = 8.1 Hz, 1H), 6.94 (d, J = 7.5 Hz, 1H), 4.00 (s, 3H), 1.91–1.89 (m, 1H), 1.41 (s, 9H), 1.31–1.29 (m, 2H), 1.06–1.02 (m, 2H).

*4-[[1-(4-tert-Butylphenyl)-2-cyclopropyl-benzimidazol-4-yl]carbamoyl]benzoic acid (**FLG390**)*:

To a solution of **Compound 3** (59 mg, 0.13 mmol) in methanol/tetrahydrofuran (2 mL/3 mL), 1M NaOH (0.39 mL, 0.39 mmol) was added at room temperature and the mixture was stirred for 15 h at room temperature. The reaction mixture was neutralized with 1M HCl and evaporated down. The residue was extracted with ethyl acetate. The combined extracts were washed with H_2O and brine, dried over $MgSO_4$ and concentrated under reduced pressure. The residue was purified by silica gel column chromatography to give **FLG390** in 79% yield. Rf = 0.35 (CH_2Cl_2/MeOH = 9/1), ^{1}H NMR (600 MHz, DMSO-d_6): δ 13.30 (brs, 1H), 10.04 (s, 1H), 8.12 (s, 4H), 7.73 (d, J = 7.7 Hz, 1H), 7.70 (d, J = 8.3 Hz, 2H), 7.53 (d, J = 8.2 Hz, 2H), 7.17 (t, J = 8.0 Hz, 1H), 6.95 (d, J = 8.0 Hz, 1H), 1.88–1.86 (m, 1H), 1.38 (s, 9H), 1.17–1.16 (m, 2H), 1.03–1.02 (m, 2H). HR-ESI-MS $[M+H]^+$ 454.21486 for $C_{28}H_{28}N_3O_3$ (calculated 454.21307). Purity (RP-HPLC): Retention time:17.3 min, 98.4%.

1. Yamashita, Y., Gohda, K.; Iguchi, Y., Fujimori, K., Oda, K., Masuda, A., Une, M., Teno, N. Discovery of FXR/PPARγ dual partial agonist. *Bioorg. Med. Chem.* **2023**, *85*, 117238, doi: 10.1016/j.bmc.2023.117238

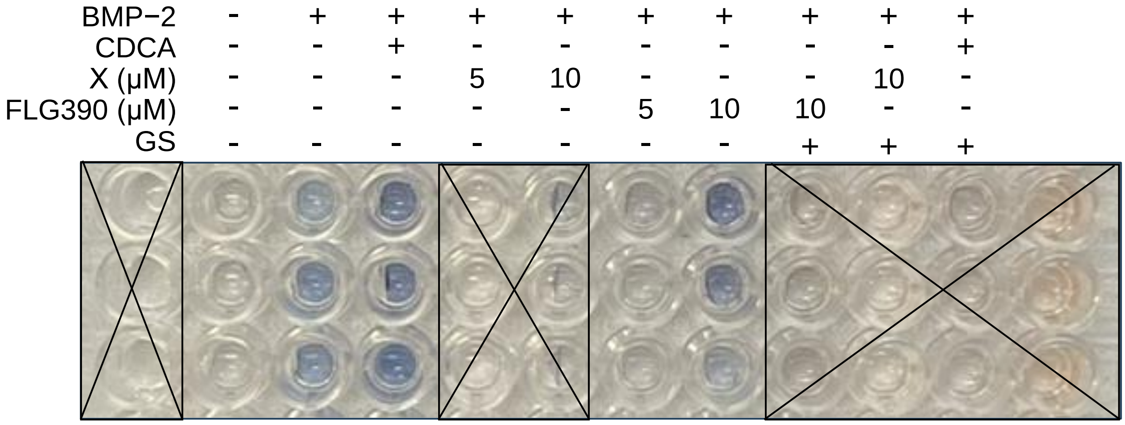

Figure S1. Uncropped data of ALP staining shown in Figure 2B.

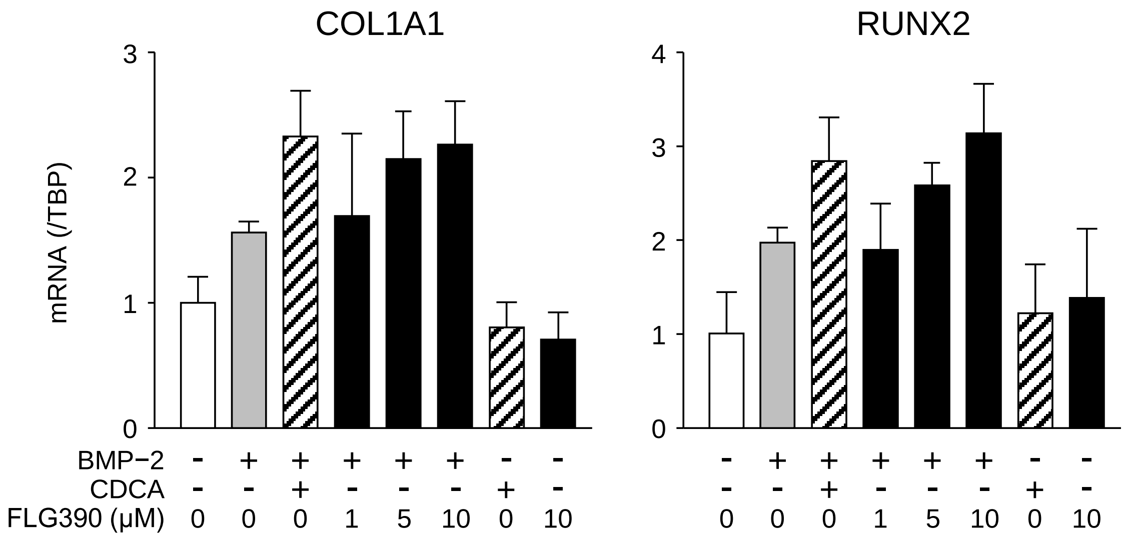

Figure S2. Expression of osteogenesis-related gene in the FXR agonist-treated ST-2 MSCs. The cells were differentiated as stated in the legend of Figure 2D. The data are presented as the mean ± S.D. from three independent experiments. *$p<0.05$.

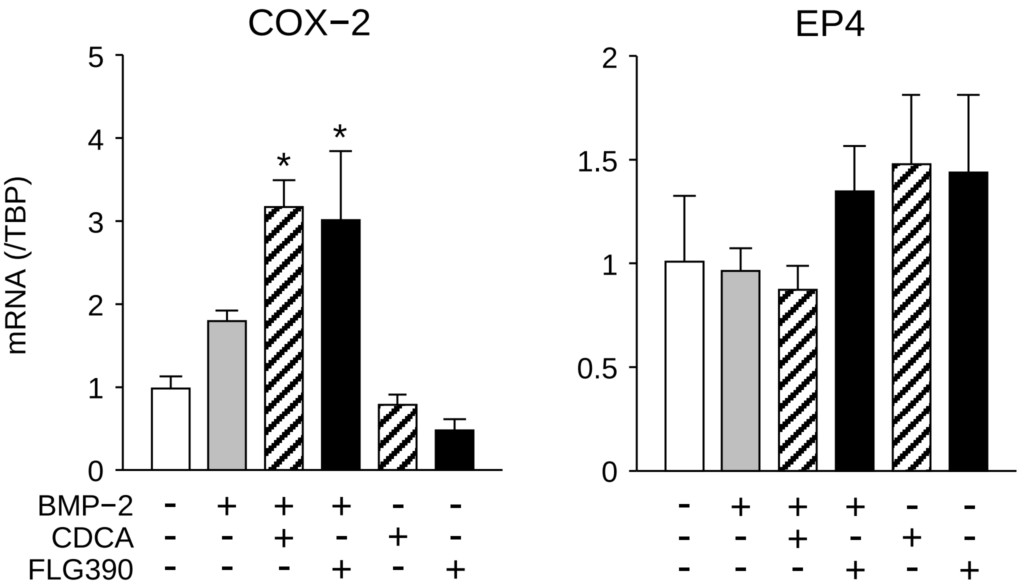

Figure S3. Expression of osteogenesis-related gene in the FXR agonist-treated ST-2 MSCs. The cells were differentiated into osteoblasts for 3 h in the medium containing each of BMP-2 (50 ng/mL; gray column), CDCA (10 μM; hatched column), and **FLG390** (10 μM; black columns). The data are presented as the mean ± S.D. from three independent experiments. *p<0.05.
